# Removal of Diverse and Abundant ARGs by MF-NF Process from Pig Manure and Digestate

**DOI:** 10.3390/membranes12070661

**Published:** 2022-06-27

**Authors:** Prantik Samanta, Harald Horn, Florencia Saravia

**Affiliations:** 1 DVGW-Research Center at the Engler-Bunte-Institut, Water Chemistry and Water Technology, Karlsruhe Institute of Technology, Engler-Bunte-Ring 9, 76131 Karlsruhe, Germany; harald.horn@kit.edu (H.H.); saravia@dvgw-ebi.de (F.S.); 2 Karlsruhe Institute of Technology, Engler-Bunte-Institut, Water Chemistry and Water Technology, Engler-Bunte-Ring 9, 76131 Karlsruhe, Germany

**Keywords:** antibiotic resistance genes, antimicrobial resistance, microfiltration, nanofiltration, manure, digestate

## Abstract

Antimicrobial resistances are emerging as one main threat to worldwide human health and are expected to kill 10 million people by 2050. Intensive livestock husbandry, along with biogas digestate, are considered as one of the biggest ARG reservoirs. Despite major concerns, little information is available on the diversity and abundance of various ARGs in small to large scale pig farms and biogas digestate slurry in Germany, followed by their consequent removal using microfiltration (MF)–nanofiltration (NF) process. Here, we report the identification and quantification of 189 ARGs in raw manure and digestate samples, out of which 66 ARGs were shared among manures and 53 ARGs were shared among both manure and digestate samples. The highest reported total ARG copy numbers in a single manure sampling site was 1.15 × 10^8^ copies/100 µL. In addition, we found the absolute concentrations of 37 ARGs were above 10^5^ copies/100 μL. Filtration results showed that the highly concentrated ARGs (except aminoglycoside resistance ARGs) in feed presented high log retention value (LRV) from 3 to as high as 5 after the MF-NF process. Additionally, LRV below 2 was noticed where the initial absolute ARG concentrations were ≤10^3^ copies/100 μL. Therefore, ARG removal was found to be directly proportional to its initial concentration in the raw manure and in digestate samples. Consequently, some ARGs (tetH, strB) can still be found within the permeate of NF with up to 10^4^ copies/100 μL.

## 1. Introduction

The biggest threat to lifesaving antibiotic therapies is the spreading and aggregation of antibiotic resistance genes (ARGs) into multidrug resistance pathogens [1,2]. Antibiotic resistance kills an estimated 700,000 people/year and it is to be expected to reach 10 million by 2050 [3]. Truly, the use of antibiotics in humans and animals largely caused the ARG reservoir in the environment [4]. Especially, manure is considered as a major source of antimicrobial pollution which is caused by the overuse of antibiotics mostly in livestock husbandry, followed by turning farms into ARG reservoirs [5,6].

Most veterinary antibiotics are poorly absorbed by the animals and consequently, a large part of it is excreted [7,8], which then unfortunately spreads within soils [9], surface water [10], and groundwater [11,12], when manure is applied as a fertilizer for its nutrient recycling practice. Moreover, antibiotic-resistance traits in manure increases by the substantial use of a subtherapeutic level of antibiotics in animal feed itself [13,14]. In addition, anaerobic digestion, which is used as one of the primary treatment processes for the excrements of intensive livestock farms [15], is suspected to even increase some of the ARG concentrations [16]. Therefore, the usage of antibiotics in farms often correlates with the expansion of the related ARGs in human pathogens, as well as the reason behind the spreading of animal antibiotic resistant bacteria (ARB) to human ARB [17,18,19]. Hence, antibiotic resistance is declared as a global public health challenge which calls for immediate steps to stop its further spreading [20,21,22].

So far, usage of multiple techniques, such as anaerobic treatment [23,24], coagulation [25], advanced chemical oxidation [26,27], and membrane bio-reactor [28] have been reported as ARG removal processes of various streams. However, the cost of these treatment processes was estimated to be high due to the large usage of reagents and they can be detrimental by bringing secondary pollutants as well. Moreover, these techniques are not feasible for direct treatment of raw manure or digestate.

Recent studies reported membrane filtration processes, such as ultrafiltration (UF), nanofiltration (NF), and reverse osmosis (RO), as an effective ARG removal process [29,30]. Membrane filtration processes are also heavily applied as a manure and digestate treatment process. Microfiltration is reported in multiple articles to be used as an effective solid–liquid separator of manure and digestate [31,32], where both fractions have the potential to further be processed to generate bio-fertilizers [33]. However, ARG removal efficiency by MF is poor and limited mostly to the intracellular ARGs [29,34,35]. In addition, the liquid fraction after solid–liquid separation is enriched with ammonium nitrogen [36], which was found to be one of the major reasons, alongside dissolved oxygen, to exhibit the strongest correlation with high ARG concentration and horizontal gene transfer [37]. Hence, further treatment of MF permeate is an absolute necessity. Therefore, additional usage of NF and RO would not only enhance the ARG removal efficiency [30,35], but also these would (a) reduce the volume, (b) produce a nitrogen-rich concentrate stream for using it as a direct fertilizer, and (c) generate a purified stream to be further used in irrigation [33,38,39,40,41,42].

To date, a few investigations have been undertaken checking the efficiency of ARG removal using NF and RO processes by treating livestock waste [35], swine wastewater [30], and reclaim water [29]. However, no studies have reported on the MF-NF treatment process of raw manure and digestate for the elimination of ARGs. Therefore, the objectives of this research study are (i) the identification and consequent quantification of diverse and abundant ARGs in raw pig manure and biogas digestate samples, followed by (ii) their removal using the MF-NF filtration process.

## 2. Materials and Methods

### 2.1. Pig Manure and Digestate Sample Collection

Pig manure samples were collected freshly in November 2020 from the pits of sampling sites 1 and 2 which are located in the state of Baden Württemberg, Germany. The digestate sample was collected in October 2020 from sampling site 3 which is located in the state of Lower Saxony, Germany. The samples were collected in 10 L canisters and quickly stored at 4 °C in the dark for further experiments.

### 2.2. Characteristics Analysis

Total suspended solid (TSS) and volatile suspended solid (VSS) measurements were completed as per APHA AWWA [43]. Chemical oxygen demand (COD), ammonium nitrogen (NH_4_^+^-N), and total phosphate (PO_4_^−3^) were measured by test method of Hach Lange GmbH. pH values of pig manure and digestate were measured by using a portable WTW ProfiLine 3110 pH meter. Dissolved total carbon (DTC), dissolved organic carbon (DOC), and dissolved total nitrogen (DTN) measurements were conducted using a Shimadzu Total Carbon Analyzer TOC-5000. Acetic acid and potassium concentrations were measured using an 881 Compact IC pro (Metrohm, Herisau, Switzerland) ion chromatograph and an inductively coupled plasma optical emission spectrometer (Agilent Technologies, ICP-OES 5110, Waldbronn, Germany), respectively. Detailed characteristics of the manure and digestate samples are provided in the supporting information (Table S1).

### 2.3. Filtration Protocols

#### 2.3.1. Pre-Treatment

Manure and digestate samples were initially sieved through a 1 mm sieve to eliminate particles from them. The samples were then prefiltered in a dead end stirred cell membrane filtration system, manufactured by Merck KGaA Germany (Supplementary Materials Figure S1), by using 0.45 µm pore sized microfiltration (MF) membranes to eliminate the suspended solids. The MF membrane characteristics are mentioned in a previous research study by Wei, Laborie, Aim, and Amy [44]. The internal membrane diameter was 14 cm, and the effective membrane area was calculated as 154 cm² in the filtration cell. Initial feed volume of 600 mL was introduced in the feed tank for MF experiments. The filtrations were then performed by applying 1 bar pressure (N2 gas, air liquid) and the rotational speed was maintained at 400 rpm. Consequently, 300 mL of permeate was collected in a sterile vial. The temperature was 25 °C ± 1 during the prefiltration experiments. ARG concentrations in feed samples were measured before each MF experiment.

#### 2.3.2. Nanofiltration

Permeate volumes of 300 mL from MF experiments were used as the feed volume for the following nanofiltration (NF) experiments which were completed by using a NF270 (DuPont, Hamm, Germany) membrane in the same stirred cell dead end filtration set up as mentioned in Section 2.3.1. Consequently, 180 mL of permeate was collected in a sterile vial after each NF experiment. Detailed characteristics of NF270 membranes are summarized in previous studies [45,46]. The NF experiments were performed at 6.5 bar as the system could sustain a maximum of 7 bar pressure. The rest of the filtration conditions were kept the same as the MF experiments. Similarly, pure water (MilliQ water, Millipore, Burlington, MA, USA) flux (PWF) was measured at 6.5 bar pressure before and after each NF experiment. The NF permeate samples were additionally analyzed for ARG concentration measurements.

### 2.4. DNA Extraction

Total DNA was extracted from each sample using a DNeasy PowerSoil Pro Kit (Qiagen Sciences, Hilden, Germany) by following the manufacturer’s instructions. Quality of DNA and its concentration were determined using a NanoDrop ND-1000 spectrophotometer (Thermo Scientific, Waltham, MA, USA).

### 2.5. Smart Chip qPCR Analysis Description

The presence and abundance of antibiotic resistance genes (ARGs) and the 16S rRNA gene in each sample were analyzed using customized primer sets [47] in a high throughput method, SmartChip qPCR system. Several primer sets were designed to target sequence diversity within the gene target to more specifically assess the environmental resistome; therefore, each primer set was analyzed independently. The threshold cycle (CT) of 27 was used as the detection limit [48,49,50,51]. Melting curve analysis and PCR efficiency were performed on all samples for each primer set. Amplicons with unspecific melting curves and multiple peaks based on the slope of melting profile were considered to be false positives and discarded from the analysis.

Briefly, the SmartChip has 5184 reaction wells with a volume of 100 nL and filled using the SmartChip Multisample Nanodispenser. qPCR cycling conditions and initial data processing was completed as previously described in [50]. qPCR reagents recommended by the manufacturer were used. Mean CT of three technical replicates in each qPCR reaction was used to calculate the ΔCT values, unless the genes were detected in only one of the three technical replicates, in which case they were removed. The 2^−ΔCT^ method (where ΔCT = CT detected gene—CT 16S rRNA gene) was used to calculate the relative abundances of the detected gene in proportion to the 16S rRNA gene in each sample [52].

### 2.6. ARG Retention Calculations

The log retention value of ARGs was calculated by following Equation (1) [30,34].

(1) $$\mathrm{Log}\;\mathrm{retention}\;\mathrm{value}\;\left(\mathrm{LRV}\right)=\mathrm{Log}\;\left(\frac{\mathrm{RS}}{\mathrm{NFP}}\right)$$

where RS referred to absolute ARG copy numbers per 100 µL in the raw manure and digestate samples and NFP referred to the ARG gene copy numbers per 100 µL in the nanofiltration permeate samples.

## 3. Results and Discussion

### 3.1. Presence of Diverse ARGs in Pig Manure and Digestate

**Figure 1:**
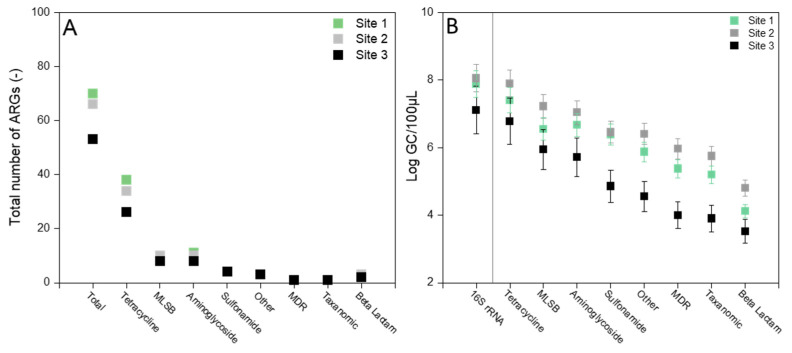
Comparison of (**A**) total detected ARGs and (**B**) log value of ARG copy numbers among three sampling sites, conferring resistance to different antibiotics, where GC referred to gene copy numbers.

In total, 189 ARGs were detected from all raw samples (Figure 1A), among which 66 ARGs were shared among manure and 53 ARGs were shared among both manure and digestate samples (Supplementary Materials, Figure S2). Antibiotic deactivation was the main resistance mechanism confined to the detected ARGs, followed by cellular protection and efflux pumps. These samples contained ARGs conferring most dominantly resistance to tetracycline (51.9%), aminoglycoside (15.3%), and MLSB (macrolide-lincosamide-streptogramin B, 14.8%) antibiotics, followed by sulfonamide (6.3%), other groups (4.8%), ß-Lactam (3.7%), taxonomic (1.6%), and MDR (multiple drug resistance, 1.6%) (Figure 1A). Similarly, the log value of total ARG copy numbers per 100 µL conferring resistance to tetracycline was found highest followed by MLSB and aminoglycoside, respectively (Figure 1B). Despite the presence of multiple ARG resistance to ß-Lactam, the log value of total ARG copy numbers conferring resistance to MDR and taxonomic were noticed 20% and 16% higher than ß-Lactam, respectively (Figure 1B). Manure samples (of sites 1 and 2) contained one to two orders of magnitude higher copy numbers of ARGs than the digestate sample (of site 3). However, the pattern of ARG copy numbers conferring resistance to different antibiotic groups was noticed similar in both manure and digestate samples (Figure 1B).

Pu, Liu, Ding, Sun, Yu, Chen, Ren, and Gong [53] found 83 shared ARGs in pig manure and digestate samples. Similarly, the most dominant types of ARGs were noticed conferring resistance to tetracycline (25–38%), aminoglycoside (20–29%), and MLSB (14–20%). Later, Zhang, Liu, Wang, Fang, Sun, Liu, and Liao [54] confirmed the findings, where the resistance of 658 ARG subtypes belonged to the most frequent classes of the above-mentioned group of antibiotics as well. A probable reason was directed to the high usage of these antibiotics in pig production [55]. The substantial presence of ARG resistance to tetracycline in pig manure has been mentioned in research studies since 2002 [56,57]. Additionally, recent studies reported more variants of it [5,58]. Consequently, Zhu, Johnson, Su, Qiao, Guo, Stedtfeld, Hashsham, and Tiedje [51] reported frequent occurrence of aminoglycoside resistance ARGs in pig manure samples. Later, Luo, Li, Li, Zhang, and Angelidaki [59] found 10 subtypes of aminoglycoside resistance ARGs and 4 subtypes of MLSB resistance ARGs in both pig manure and digestate samples. It is presumed that not only the antibiotics but also the striking number of additives used increase the prospect of coresistance in genetic elements [60].

### 3.2. Absolute ARG Abundances in Raw Manure and Digestate

**Figure 2:**
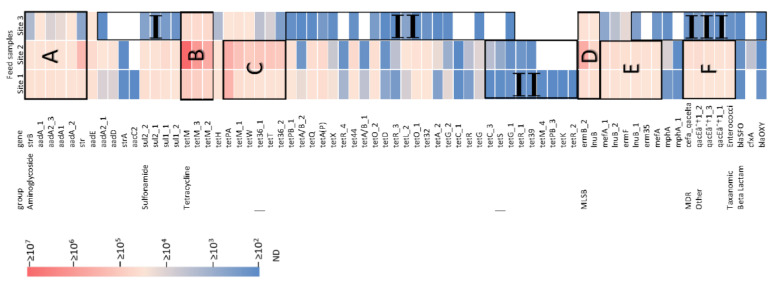
Absolute ARG concentration (per 100 µL) profile, resistance to different antibiotic groups in each sampling site. Zones (A), (B), and (D) are enriched in all sampling sites; (C), (E), and (F) are enriched in sites 1 and 2 but not in site 3; Zones I, II, and III denoted the absolute ARG copy numbers ≤ 10^3^ per 100 µL.

Pig manure and digestate samples were highly enriched with ARGs. The total ARG copy number was highest in the pig manure sample of site 2 (1.15 × 10^8^ copies), which was one and two orders of magnitude higher than sites 1 and site 3, respectively (Figure 1B). Absolute concentrations of 37 ARGs were found above 10^5^ copies per 100 µL. The absolute ARG concentrations in the raw manure and digestate samples indicated the actual ARG copy numbers per 100 µL (Figure 2). High enrichment of ARGs in all samples demonstrated the substantial expansion of an antibiotic resistance reservoir in the sampling sites, including the enrichment of up to 38 *tet* genes in a single site, followed by 11 and 10 aminoglycoside and MLSB resistance genes, respectively (Supplementary Materials Figure S2).

In the digestate sample of site 3, 75% of the aminoglycoside resistance genes were found above 10^4^ copies per 100 µL. Although, it was reduced to 38% for *tet* genes. Consequently, 24% of the *tet* genes were found as low as 10^3^ copies per 100 µL in the manure sample of site 1 and 10% of the *tet* genes were not detected in the manure sample of site 2. On the contrary, all ARG resistance to sulfonamide, MDR, other, and taxonomic groups were detected above 10^5^ copies per 100 µL in manure samples. In addition, beta lactam resistance genes were nearly not detectable in all samples. In general, the lowest number of ARGs was detected in the digestate sample of site 3. Their average concentration was 10^4^ copies per 100 µL, which was one to two orders of magnitude lower compared to the average ARG presence in manure samples.

This result not only informs the extension of the antimicrobial reservoir in large to medium sized livestock husbandry and biogas plants but also shows the substantial abundances of the detected ARGs, which may lead to possible horizontal transfer in the environment [51]. The various sets of detected ARGs were potentially resistant to all major classes of antibiotics, including critically important antibiotics for human medicines, such as tetracycline, macrolides, and aminoglycoside [61]. Looft, Johnson, Allen, Bayles, Alt, Stedtfeld, Sul, Stedtfeld, Chai, and Cole [13] detected 57 ARGs from the manure of selected pigs, out of which 8 ARGs were enriched. Later Zhu, Johnson, Su, Qiao, Guo, Stedtfeld, Hashsham, and Tiedje [51] demonstrated the list of 62 ARGs which were frequently detected in multiple animal farms. However, the maximum enrichment of an ARG in a single site was reported as 90,000 copies per mL. This is strikingly three orders of magnitude lower than the highest enrichment of the ARG (*tetM*) found in the present study. However, a few recent studies which focused on some particular *tet* and *sul* genes have reported the highest enrichment from 10^6^ to even 10^11^ copies per mL [29,30], which is in accordance with the results presented here.

The average enrichment (10^4^ copies per 100 µL) of aminoglycoside resistance ARGs compared to the other antibiotic groups was noticed as highest in the digestate sample of site 3. It is comparable with the previous findings by Pu, Liu, Ding, Sun, Yu, Chen, Ren, and Gong [53]. However, depending on the anaerobic digestion conditions, the results could be turned around [62,63]. In previous studies, it was observed that aminoglycoside resistance ARGs were coenriched due to their probable aggregation in the mobile genetic elements [13,64,65,66,67]. Consequently, Binh, Heuer, Kaupenjohann, and Smalla [68] mentioned that the presence of them in the integrons may cause their enrichment as well. The presence of sulfonamide resistance ARGs in manure and digestate samples are ubiquitous and are among the most enriched ones [51,53]. The enrichment of *sul1* and *sul2* subtypes in the digestate sample of site 3 were two orders of magnitude lower than the manure samples. This could be the result of the alteration of the digestate feed itself from raw pig manure to a waste mix. This is in accordance with a previous study, where Song, Wang, Gu, Zhang, Yin, Li, Qian, and Sun [69] revealed that the usage of pig manure and wheat straw mixture as digestate feed instead of raw pig manure resulted in lower *sul1* and *sul2* concentrations in the digestate.

However, other studies found that the digestion operation at 35 °C may increase sulfonamide resistance ARG concentration in the digestate [70,71]. Hence, further studies are required to clarify the ambiguity of the enrichment of the sulfonamide resistance genes in digestate. Zhu, Johnson, Su, Qiao, Guo, Stedtfeld, Hashsham, and Tiedje [51] detected 22 *tet* genes, which were shared in pig manure samples of multiple animal farms. They reported *tetQ*, *tetW*, *tetX*, *tet*(*32*), *tetO*, *tetM*, *tetL*, and *tetG* as the most abundant *tet* genes. We detected 38 *tet* genes in a pig manure sample of a single sampling site (site 1) where the most abundant *tet* genes were *tetM*, *tetH*, *tetW*, *tetT*, *tetQ*, and their subtypes which are in accordance with the findings by Zhu, Johnson, Su, Qiao, Guo, Stedtfeld, Hashsham, and Tiedje [51]. The 73% increased number of *tet* genes in our study compared to the previous literature reflected our detailed sampling process and the precise ARG detection method.

Although swine farms are considered as the hotspots of antimicrobial resistance for antibiotic-free [13,72] and antibiotic-treated animals [73,74], the enrichment of the ARGs found in this study was on par with previous literature. In addition, Pärnänen, Narciso-da-Rocha, Kneis, Berendonk, Cacace, Do, Elpers, Fatta-Kassinos, Henriques, and Jaeger [75] gathered a large number of ARG datasets from the different geographic regions of Europe and found that, in Germany, the abundances of gene families resistant to tetracycline and MLSB antibiotics was higher than other antibiotic groups. In particular, MLSB resistance *ermF* and aminoglycoside resistance *aadA* genes were noticed with very high prevalence. Both findings are in accordance with the present study.

The specificity of the diverse ARGs and their fate in raw and treated samples reflected the influence of antibiotics, particularly the residues [76,77,78,79]. The alarming enrichment of ARGs at farm level might exhibit the threat to the human population by getting transferred from livestock animals to human-related bacteria [18,80].

### 3.3. Removal of ARGs from Raw Manure and Digestate by Nanofiltration

The World Health Organization (WHO) referred to antimicrobial resistance as the emerging threat to the treatment against infections caused by parasites, viruses, and bacteria in their Global Report on Surveillance [81]. The substantial use of antibiotics made the presence of resistant genes and the mobile genetic elements ubiquitous in all possible environments. In particular, livestock husbandry was identified as the antimicrobial reservoir which has the potential to spread ARGs and the mobile genetic elements into the environment [58,70,82]. The concerns may hinder the further reuse of manure and digestate in agriculture. Hence, a suitable antimicrobial resistance removal technology is the need of the hour.

Membrane filtration processes, including MF to RO, have been applied as effective processes to remove ARGs from pig wastewater and digestate in recent years [30,35,83]. MF is widely used as a solid–liquid separation process for manure and digestate treatment [31,32]. Therefore, in this study, MF of raw pig manure and digestate was initially used to remove suspended solids from it. Although, a previous study stated that MF could separate ARGs only to some extent [28], especially the intracellular ARGs by removing almost all bacteria (typically 0.5–5.0 µm) [30]; however, the absolute concentration difference between the feed and the permeate after MF remained within one to two orders of magnitude [29]. In addition, Gros, Marti, Balcázar, Boy-Roura, Busquets, Colon, Sanchez-Melsio, Lekunberri, Borrego, and Ponsá [35] noticed no difference in *tetW* concentration between solid and liquid fractions of livestock waste and no retention of *ermT*, *qnrA,* and *qnrB* by MF was observed by Lu, Zhang, Wu, Wang, and Cai [29] using MF membrane. Most importantly, MF could not retain extracellular or free DNA, which could result in the dissemination of the ARGs, that are encoded within this DNA, into the soil and aquatic environment [34]. In this work, the MF permeates were directly filtered by NF membrane to minimize the further transmission probability, followed by detecting ARGs in the NF permeate to evaluate the final retention using the MF-NF process.

The highly concentrated ARGs in raw samples are presented in zones A to F and the low concentrated ARGs in raw samples are presented in zones I to III of Figure 2. Similarly, the LRV of these highly concentrated ARGs after the MF-NF process are presented in zones A to F and the LRV of the low concentrated ARGs after the MF-NF process are presented in zones.

**Figure 3:**
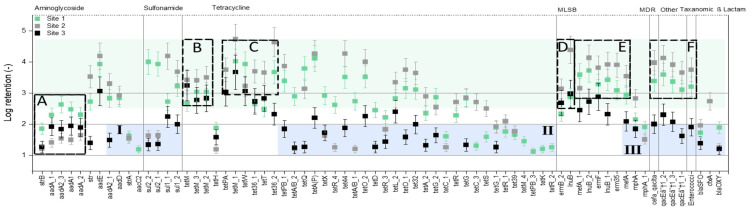
Log retention values (LRVs) of individual ARGs of pig manure and digestate samples of sampling sites 1, 2, and 3. Zones (A), (B), and (D) were enriched in raw samples of all the sampling sites; (C), (E), and (F) were enriched in the raw samples of sites 1 and 2 but not in the raw digestate sample of site 3; Zones I, II, and III denoted the absolute ARG copy numbers ≤10^3^/100 µL in the raw samples of all sampling sites.

I to III of Figure 3. Apart from the highly concentrated aminoglycoside resistance genes (Figure 2, Zone A), all other ARG-enriched zones (Figure 2, Zones B to F) showed LRV from 3 (99.9%) to as high as 5 (99.999%) after the MF-NF process (Figure 3, Zones B to F). Consequently, LRV ≤ 2 (≤99%) was noticed in Zones I, II, and III of Figure 3, where the initial absolute ARG concentrations were ≤10^3^ copies per 100 µL (Figure 2, Zones I, II, and III). In addition, ARG removal was found to be directly proportional to its initial concentration in the feed apart from mostly aminoglycoside resistance and a few tetracycline resistance genes (Supplementary Materials, Figure S3A–C).

LRVs were calculated by following Equation (1). Despite being enriched with ARGs in all samples, the average LRV remained only 1.5 for aminoglycoside resistance genes, represented in Zone A of Figure 3. The lowest LRV was found to be 1.2 for *strB* in the manure sample of site 2 and in the digestate sample. Pärnänen, Narciso-da-Rocha, Kneis, Berendonk, Cacace, Do, Elpers, Fatta-Kassinos, Henriques, and Jaeger [75] gathered a large number of ARG datasets from various European countries and noticed the persistence of aminoglycoside resistance ARGs (*aadA* and *strB*) after treatment in more than 90% of the samples. In addition, Gros, Marti, Balcázar, Boy-Roura, Busquets, Colon, Sanchez-Melsio, Lekunberri, Borrego, and Ponsá [35] mentioned that the ARGs with low retention after RO were directly linked with class I integrons [84,85,86]. Hence, the similarities in low retention of aminoglycoside resistance genes in this study might be attributed to their linkage in class I integrons [68] as well. The LRV of sulfonamide resistance genes (subtypes of *sul1* and *sul2*) was 4 in manure samples. Lan, Kong, Sun, Li, and Liu [30] reported that the LRVs of *sul1* and *sul2* genes were 5.29 and 6.13, respectively, after the NF process. High initial concentration of ARGs was found as the key reason for this very high retention. Similarly, Lu, Zhang, Wu, Wang, and Cai [29] found that the LRVs of *sul1* and *sul2* were 2.8 and 3.3, respectively, after RO filtration. High efficiency of eDNA removal by RO was mentioned as a major reason for higher removal. These findings are in accordance with the present study. However, LRVs of *sul_1* and *sul_2* in the digestate sample of site 3 were 1.32 and 1.36, respectively. Interestingly, the lower retention could directly be linked to the lower initial concentration of the genes in Figure 3, Zone I. In addition, Gros, Marti, Balcázar, Boy-Roura, Busquets, Colon, Sanchez-Melsio, Lekunberri, Borrego, and Ponsá [35] linked the low retention of *su1* after RO with their linkage to class I integrons. A similar trend was noticed for *tet* genes as well. Highly enriched *tet* genes such as *tetM*, *tetW*, *tetT*, and their subtypes in zones B and C were also retained efficiently after the NF process. The maximum LRV of *tetM*, *tetW*, and *tetT* genes were 3.46, 4.72, and 3.93, respectively. These results are in accordance with previous studies, where the max LRV of *tet* genes after NF and RO was reported between 2.5 and 7.84 [29,30,35]. However, the LRVs of *tet* genes with low initial concentration (≤ 10^3^ copies per 100 µL) were below 2 (Figure 3, Zone II). In the manure of site 1, the lowest retention was noticed for *tetPB_3*, *tetK,* and *tetR_2* genes. Their LRVs were between 1.11 and 1.25. The similar range of LRVs was noticed for the lowest retained *tetR_4* and *tetA/B_1* genes in the manure sample of site 2 and *tetA/B_2* and *tetQ* genes in the digestate sample of site 3. Lu, Zhang, Wu, Wang, and Cai [29] noticed low LRV of 1.6 of the *tetB* gene after RO filtration. Interestingly, they mentioned that the initial concentration of the *tetB* gene before RO filtration was below 10^3^ copies. This is in accordance with the present study. Furthermore, the LRV between 3 and 4.41 was noticed for MLSB, MDR, other, and taxonomic resistance genes in zones D, E, and F of Figure 3 which were enriched in raw manure and digestate samples (Figure 2, Zones D, E, and F). The LRVs above 2.3 for MLSB resistance *erm* genes after RO filtration were reported in previous studies [29,35], which is in accordance with this study. Subsequently, the LRVs of ß Lactam resistance genes in manure and digestate samples were below 2. The lowest LRV of 1.18 was noticed for *bla*OXY in the manure of site 2 and in the digestate sample. Cristóvão, Tela, Silva, Oliveira, Bento-Silva, Bronze, Crespo, Crespo, Nunes, and Pereira [87] noticed only 90.59% removal of the *bla*NDM gene after NF with Desal 5 DK membrane. However, the removal rate of other *bla* (*bla*KPC, *bla*OXA-48 and *bla*VIM) genes were reported above 99.6%. Dissemination of aerosol near the sampling point was mentioned as the reason for low ARG presence in the NF permeate. In summary, the LRV of enriched genes after the NF process was higher than 3 to as high as 5. However, the retention of genes with low initial concentration remained below 99% (LRV 2).

A size exclusion mechanism was previously mentioned as one of the prime reasons for ARG removal by the membrane filtration process [88,89,90,91]. A recent study by Cheng and Hong [92] assessed the sizes of the plasmids of *bla*NDM-1, *bla*CTX-M-15, and *bla*OXA-48 ARGs by dynamic light scattering technique and were noticed to be within 460–560 nm in diameter. On the other hand, the average pore diameter of NF270 was reported as 0.84 nm [93], which was 560–660 times smaller than the previously mentioned plasmid diameter. Therefore, in our study, the size exclusion of ARGs by NF270 is also considered as one of the main ARG retention mechanisms. Electrostatic charge repulsion was considered as the next major ARG retention mechanism by NF. The hydrophilicity of the extracellular plasmids is evident due the exposed sugar–phosphate bond of DNA [94]. In support, Cheng and Hong [92] also found that the zeta potential value of the above-mentioned three plasmids was greater than −22 mV. Consequently, the zeta potential of the NF270 membrane was reported as −24.7 mV at pH 8 [93]. Therefore, the electrostatic charge repulsion mechanism might play a major role in ARG retention in the present study as well. This is in accordance with Ager, Latulippe, and Zydney [95], who reported the retention of negatively charged plasmid molecules enhanced when filtering with negatively charged membranes. However, Slipko, Reif, Woegerbauer, Hufnagl, Krampe, and Kreuzinger [34] observed higher adsorption of ARGs on less charged membranes. Moreover, in our study, ARG retention was found largely proportional to its initial enrichment in the feed. It is in accordance with findings of Slipko, Reif, Woegerbauer, Hufnagl, Krampe, and Kreuzinger [34]. They hypothesized that the free DNA molecules, which adsorb on the membrane surface, subsequently blocked the passage through membrane, followed by reduction in ARG permeation. Similar findings were noticed by Lan, Kong, Sun, Li, and Liu [30], where extremely high level presence of *sul* and *tet* genes in raw swine wastewater leads to LRV of 4.98–9.52 after NF and RO treatment of the sewage. Lastly, the interaction of free DNA molecules with manure and digestate matrix might serve as an additional ARG removal mechanism by NF270 [34,89].

Multiple studies reported the complete removal of ARGs by RO, NF, and UF application, especially in case of wastewater post treatment [96,97]; however, we observed some ARGs (e.g., *tetH*, *strB*, etc.) were present at a concentration of 10^3^ to 10^4^ copies per 100 µL in NF permeate. This in accordance with the findings where nearly the same ARG concentration was observed in NF and RO permeate [29,35], when filtering livestock waste and reclaim water. According to Gros, Marti, Balcázar, Boy-Roura, Busquets, Colon, Sanchez-Melsio, Lekunberri, Borrego, and Ponsá [35], fouled membrane permeates more ARGs when compared with clean membranes. Tang, Kwon, and Leckie [98] observed that fouled NF270 membrane turned considerably less negative in the presence of DOC and calcium divalent ions. Therefore, carefully considering the manure and digestate composition where DOC concentration was 10 times higher (supporting information, Table S1), it might be hypothesized that the severe membrane fouling followed by reduced electrostatic repulsion effect may lead to the permeation of some ARGs. However, previous studies proposed that the permeation of this DNA, which was 500–600 times bigger in size compared to the membrane pores, could only be possible when the DNA could be stretched and elongated through the pores while possessing a ‘snake-like’ movement [88,90,91]. Arkhangelsky, Sefi, Hajaj, Rothenberg, and Gitis [88] found that the DNA penetration was linearly correlated to the applied pressure and was completely unaffected by its length. They observed that a critical pressure threshold of 2–3 bars must be reached to stretch out the DNA. This is in accordance with the present study, where all the NF experiments were performed at 6.5 bars. Interestingly, the latest findings of pores or voids in so called non-porous membranes [99,100] may influence the permeation of certain ARGs as well. However, this needs further investigation. The forward and reverse primer numbers of all ARGs were mentioned in the Supplementary Materials Table S2.

## 4. Conclusions

Pig manure and digestate containing abundant and diverse ARGs along with its sheer volume is considered as a major antibiotic resistance reservoir and a public health hazard. In this present study, 189 ARGs were detected from all raw samples, among which 66 ARGs were shared among manures and 53 ARGs were shared among both manure and digestate samples. The highest reported total ARG copy number in a single manure sampling site was 1.15 × 10^8^ copies. These highly alarming ARG numbers indicated the uncontrolled use of antibiotics in pig farms expanded the antimicrobial reservoir in the farm environment.

The combination of prefiltration by MF, followed by nanofiltration by NF270 membrane investigated herein, represented the suitability for raw pig manure and digestate treatment. Results indicated that various ARGs and 16S rRNA genes could effectively be removed to LRV above 3 to as high as 5 by this advanced membrane filtration process. Size exclusion and electrostatic repulsion were considered as the main ARG removal mechanisms by NF270. Interestingly, ARG removal was found directly proportional to its initial concentration in the raw manure and digestate samples. Nevertheless, some points which need further investigation are given below:

1. Further removal of some ARGs (e.g., *tetH*, *strB*) which were present at a concentration of 10^3^ to 10^4^ copies per 100 µL in NF permeate.
2. Better pretreatment of the raw manure and digestate samples for further fouling reduction of NF270.

Furthermore, the established guideline values of raw manure application as a fertilizer should be monitored rather strictly to prevent soil and groundwater antimicrobial pollution as well as their uptake by crops.

Lastly, with the rise in the antibiotic consumption in livestock production, human health is facing a bigger issue of antimicrobial resistance. Several studies have already claimed the correlation of animal farming with the rise in ARG concentration in the nearby groundwater and surface water which might result in disease outbreaks, virulence, and enhance the transmission. The present study could only raise the awareness to an elevated level by presenting the strikingly high concentration of ARGs that were found in the manure and digestate.

## Data Availability

Not applicable.

## Supplementary Materials

The following supporting information can be downloaded at: https://www.mdpi.com/article/10.3390/membranes12070661/s1, Figure S1: Stirred cell dead end membrane filtration system; Figure S2: Number of detected ARGs; Figure S3: Relation between ARG concentration in the feed and their consequent removal by MF-NF process. Every ARG is represented with a unique serial number which is mentioned in Table SD2; Table S1: Characteristics of the pig manure and digestate samples; Table S2: ARG charcteristics.
