# Supplementary material for: Removal of Diverse and Abundant ARGs by MF-NF Process from Pig Manure and Digestate
Source: Membranes (Basel). 2022 Jun 27;12(7):661. doi: 10.3390/membranes12070661 (PMC9317629; doi:10.3390/membranes12070661)
Supplement: Supplementary file 1 [file membranes-12-00661-s001.zip › membranes-1761282-supplementary.pdf]

Supporting information

Table S1. Characteristics of the pig manure and digestate samples

Parameters	Site 1<br>Pig manure	Site 2<br>Pig manure	Site 3<br>Digestate
TSS [gL^{-1}]	3	4.9	1.8
VSS [% of TSS]	83.3	78	67.8
COD [gL^{-1}]	11.3	11.8	19.5
NH_4^+ - N [gL^{-1}]	4.4	2.9	1.8
PO_4^{-3} [mgL^{-1}]	394	323	245
pH	7.8	7.8	7.8
TOC [gL^{-1}]	4.9	4.3	3.9
DOC [gL^{-1}]	3.3	3	1.8
DTN [gL^{-1}]	3	2	1.5
Acetic acid [mgL^{-1}]	3637	2211	<100
K^+ [mgL^{-1}]	1794	1698	2663

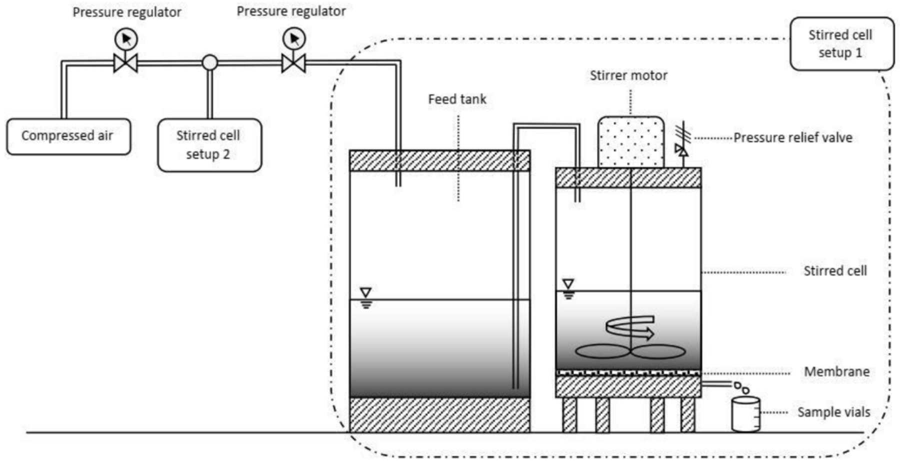

Figure S1. Stirred cell dead end membrane filtration system

Description	Total genes No.	Tetracycline	Aminoglycoside	MLSB	Sulfonamide	Other	Beta Lactam	Taxanomic	MDR
Site 1 manure	70	38	11	10	4	3	2	1	1
Site 2 manure	66	34	10	10	4	3	3	1	1
Site 3 digestate	53	26	8	8	4	3	2	1	1
Site 1 N270 perm	16	7	6	2	1	0	0	0	0
Site 2 NF270 perm	25	15	6	2	2	0	0	0	0
Site 3 NF270 perm	13	4	6	1	2	0	0	0	0

Figure S2. Number of detected ARGs.

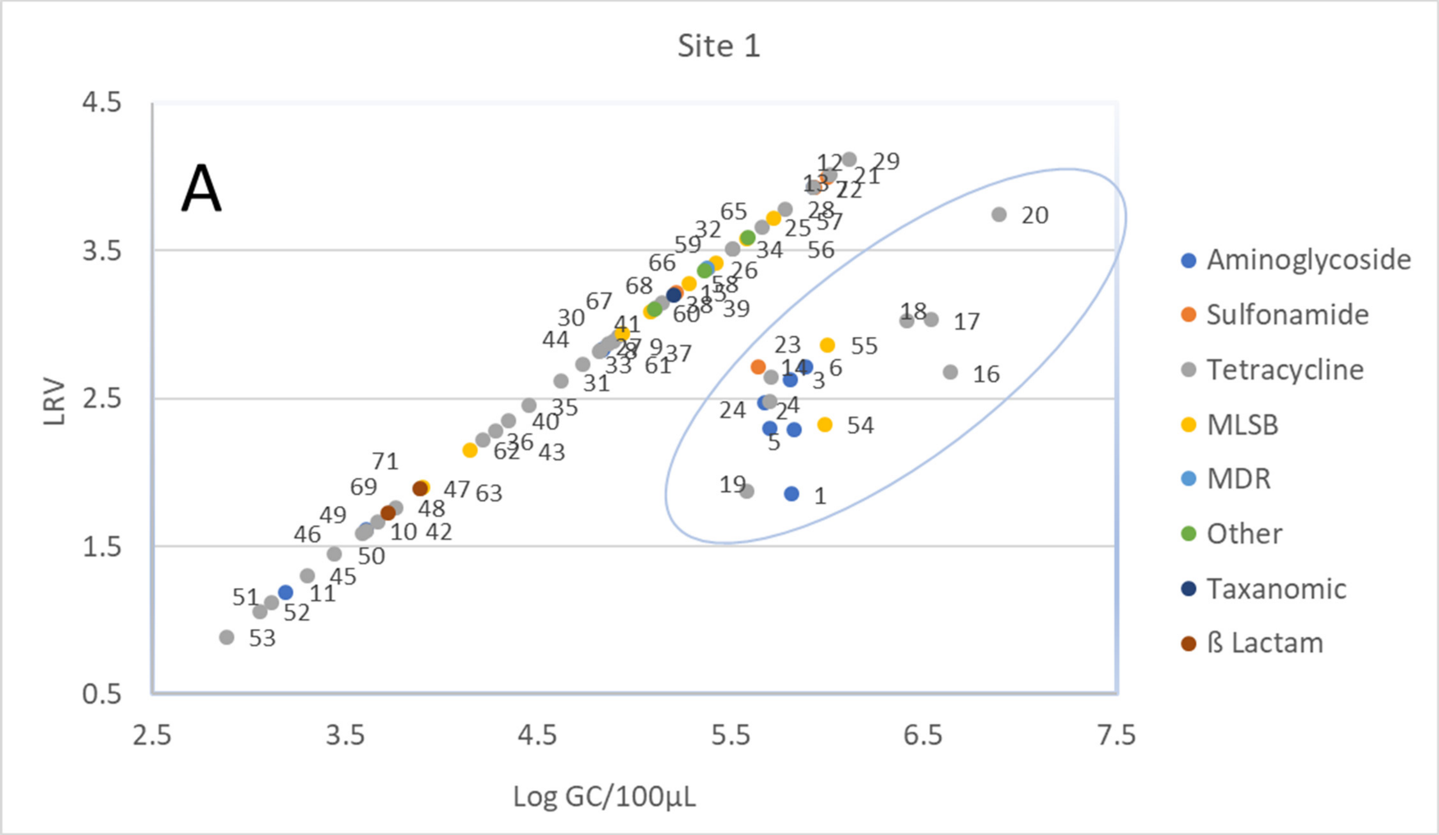

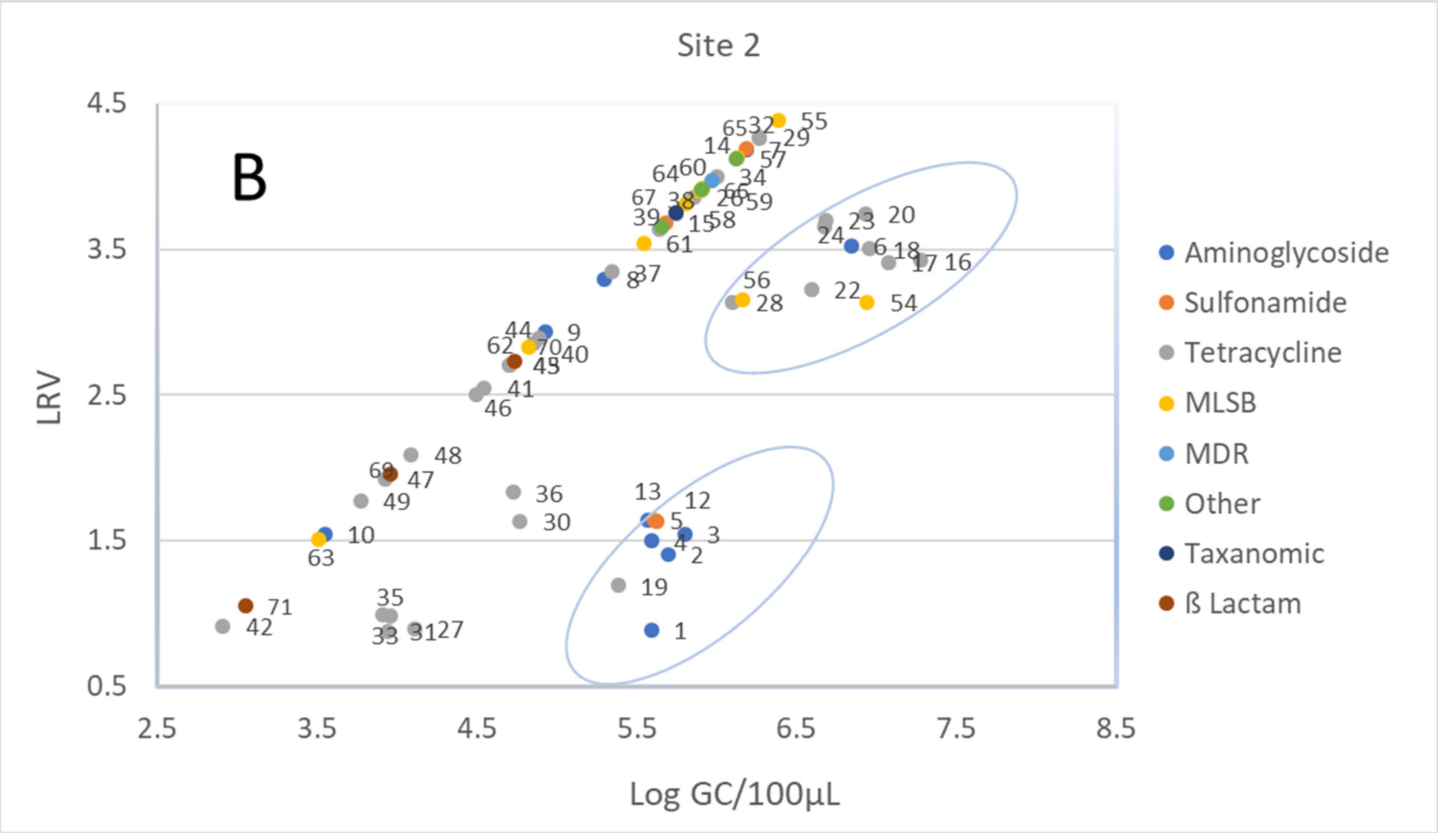

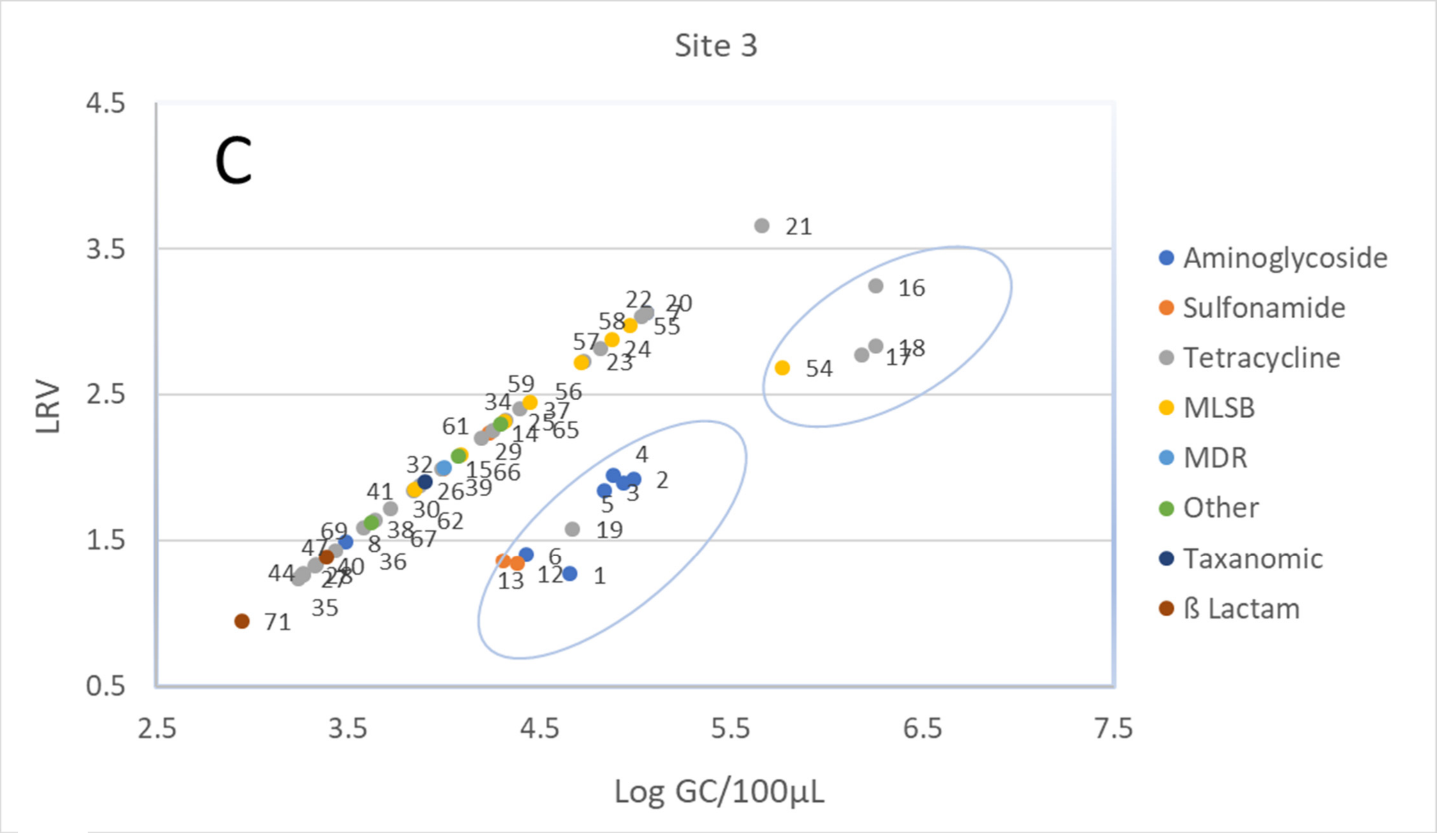

Figure S3. Relation between ARG concentration in the feed and their consequent removal by MF-NF process. Every ARG is represented with a unique serial number which is mentioned in Table SD2.

Table S2. ARG charcteristics

AB groups	SL No.	ARGs	Forward Primer	Reverse Primer
Aminoglycoside	1	strB	GCTCGGTCGTGAGAACAATCT	CAATTTCGGTCGCCTGGTAGT
	2	aadA_1	GTTGTGCACGACGACATCATT	GGCTCGAAGATACCTGCAAGAA
	3	aadA2_3	CAATGACATTCTTGCGGGTATC	GACCTACCAAGGCAACGCTATG
	4	aadA1	TGTACGGCTCCGCAGTG	CACGGAATGATGTCGTCGTG
	5	aadA_2	CGAGATTCTCCGCGCTGTA	GCTGCCATTCTCCAAATTGC
	6	str	AATGAGTTTTGGAGTGTCTCAACGTA	AATCAAAACCCCTATTAAAGCCAAT
	7	aadE	TACCTTATTGCCCTTGGAAGAGTTA	GGAACTATGTCCCTTTTAATTCTACAATCT
	8	aadA2_1	ACGGCTCCGCAGTGGAT	GGCCACAGTAACCAACAAATCA
	9	aadD	CCGACAACATTTCTACCATCCTT	ACCGAAGCGCTCGTCGTATA
	10	strA	CCGGTGGCATTTGAGAAAAA	GTGGCTCAACCTGCGAAAAG
	11	aacC2	ACGGCATTCTCGATTGCTTT	CCGAGCTTCACGTAAGCATTT
Sulfonamide	12	sul2_2	TCATCTGCCAAACTCGTCGTTA	GTCAAAGAACGCCGCAATGT
	13	sul2_1	TCCGGTGGAGGCCGGTATCTGG	CGGGAATGCCATCTGCCTTGAG
	14	sul1_1	CGCACCGGAAACATCGCTGCAC	TGAAGTTCCGCCGCAAGGCTCG
	15	sul1_2	GCCGATGAGATCAGACGTATTG	CGCATAGCGCTGGGTTTC
Tetracycline	16	tetM	GGAGCGATTACAGAATTAGGAAGC	TCCATATGTCCTGGCGTGTC
	17	tetM_3	CATCATAGACACGCCAGGACATAT	CGCCATCTTTTGCAGAAATCA
	18	tetM_2	GCAATTCTACTGATTTCTGC	CTGTTTGATTACAATTTCCGC
	19	tetH	TTTGGGTCATCTTACCAGCATTAA	TTGCGCATTATCATCGACAGA
	20	tetPA	GGAAACCTTAGTTCAGTGACTTGG	CCCATTTAACCACGCACTGAA
	21	tetM_1	TAATATTGGAGTTTTAGCTCATGTTGATG	CCTCTCTGACGTTCTAAAAGCGTATTAT
	22	tetW	ATGAACATTCCCACCGTTATCTTT	ATATCGGCGGAGAGCTTATCC
	23	tet36_1	AGAATACTCAGCAGAGGTCAGTTCCT	TGGTAGGTCGATAACCCGAAAAT
	24	tetT	CCATATAGAGGTTCCACCAAATCC	TGACCCTATTGGTAGTGGTTCTATTG
	25	tet36_2	TGCAGGAAAGACCTCCATTACAG	CTTTGTCCACACTTCCACGTACTATG
	26	tetPB_1	TGGGCGACAGTAGGCTTAGAA	TGACCCTACTGAAACATTAGAAATATACCT
	27	tetA/B_2	GCCCAGTGCTGTTGTTGTCAT	TGAAAGCAAACGGCCTAAATACA
	28	tetQ	CGCCTCAGAAGTAAGTTCATACACTAAG	TCGTTCATGCGGATATTATCAGAAT
	29	tetA(P)	AGTTGCAGATGTGTATAGTCGTAAACTATCTATT	TGCTACAAGTACGAAAACAAAACTAGAA
	30	tetX	AAATTTGTTACCGACACGGAAGTT	CATAGCTGAAAAAATCCAGGACAGTT
	31	tetR_4	CGCGATGGAGCAAAAGTACAT	AGTGAAAAACCTTGTTGGCATAAAA
	32	tet44	CTCATGTAGATGCAGGAAAGACG	GTAACTGCTGCCTGAATTGTGA
	33	tetA/B_1	AGTGCGCTTTGGATGCTGTA	AGCCCCAGTAGCTCCTGTGA
	34	tetO_2	CAACATTAACGGAAAGTTTATTGTATACCA	TTGACGCTCCAAATTCATTGTATC
	35	tetD	AATTGCACTGCCTGCATTGC	GACAGATTGCCAGCAGCAGA
	36	tetR_3	CGCGATAGACGCCTTCGA	TCCTGACAACGAGCCTCCTT
	37	tetL_2	ATGGTTGTAGTTGCGCGCTATAT	ATCGCTGGACCGACTCCTT
	38	tetO_1	ATGTGGATACTACAACGCATGAGATT	TGCCTCCACATGATATTTTTCCT
	39	tet32	CCATTACTTCGGACAACGGTAGA	CAATCTCTGTGAGGGCATTTAACA
	40	tetA_2	CTCACCAGCCTGACCTCGAT	CACGTTGTTATAGAAGCCGCATAG
	41	tetG_2	CATCAGCGCCGGTCTTATG	CCCCATGTAGCCGAACCA
	42	tetC_1	CATATCGCAATACATGCGAAAAA	AAAGCCGCGGTAAATAGCAA
	43	tetR	CCGTCAATGCGCTGATGAC	GCCAATCCATCGACAATCACC
	44	tetG	TCGCGTTCCTGCTTGCC	CCGCGAGCGACAAACCA
	45	tetC_3	TGCGTTGATGCAATTTCTATGC	GGAATGGTGCATGCAAGGAG
	46	tetS	TTAAGGACAAACTTTCTGACGACATC	TGTCTCCCATTGTTCTGGTTCA
	47	tetG_1	TCAACCATTGCCGATTCGA	TGGCCCGGCAATCATG
	48	tetR_1	CAATCCATCGACAATCAC	GACAATCAGCTACTTCAC
	49	tet39	TATAGCGGGTCCGGTAATAGGTG	CCATAACGATCCTGCCCATAGATAAC
	50	tetM_4	TGGCAAGACGAGTTTGACTGA	GATCGCTCCACTTCAGCGATAA
	51	tetPB_3	ACACCTGGACACGCTGATTTT	ACCGTCTAGAACGCGGAATG
	52	tetK	CAGCAGTCATTGGAAAATTATCTGATTATA	CCTTGTACTAACCTACCAAAAATCAAAATA
	53	tetR_2	ATGAGTTCGGCCAGAATTTCC	GGTTGTGCGCGAAATGATT
MLSB	54	ermB_2	GAACACTAGGGTTGTTCTTGCA	CTGGAACATCTGTGGTATGGC
	55	lnuB	GGATCGTTTACCAAAGGAGAAGG	AGCATAGCCTTCGTATCAGGAA
	56	mefA_1	CCGTAGCATTGGAACAGCTTTT	AAACGGAGTATAAGAGTGCTGCAA
	57	lnuB_2	AAAGGAGAAGGTGACCAATACTCTGA	GGAGCTACGTCAAACAACCAGTT
	58	ermF	TCTGATGCCCGAAATGTTCAAG	TGAAGGACAATTGAACCTCCCA
	59	lnuB_1	TGAACATAATCCCCTCGTTTAAAGAT	TAATTGCCCTGTTTCATCGTAAATAA
	60	erm35	TTGAAAACGATGTTGCATTAAGTCA	TCTATAATCACAACTAACCACTTGAACGT
	61	mefA	CCGTAGCATTGGAACAGCTTTT	AAACGGAGTATAAGAGTGCTGCAA
	62	mphA	TCAGCGGGATGATCGACTG	GAGGGCGTAGAGGGCGTA
	63	mphA_1	CTGACGCGCTCCGTGTT	GGTGGTGCATGGCGATCT
MDR	64	cefa_qacelta	TAGTTGGCGAAGTAATCGCAAC	TGCGATGCCATAACCGATTATG
Other	65	qacEΔ1_2	CCCCTTCCGCCGTTGT	CGACCAGACTGCATAAGCAACA
	66	qacEΔ1_3	GTCGGTGTTGCTTATGCAGTCT	CAACCAGGCAATGGCTGTAA
	67	qacEΔ1_1	TCGCAACATCCGCATTAAAA	ATGGATTTCAGAACCAGAGAAAGAAA
Taxanomic	68	Enterococci	AGAAATTCCAAACGAACTTG	CAGTGCTCTACCTCCATCATT
Beta Lactam	69	blaSFO	CCGCCGCCATCCAGTA	GGGCCGCCAAGATGCT
	70	cfxA	TCATTCCTCGTTCAAGTTTTCAGA	TGCAGCACCAAGAGGAGATGT
	71	blaOXY	CGTTCAGGCGGCAGGTT	GCCGCGATATAAGATTTGAGAATT
